# Supplementary material for: Characterization and structural basis of a lethal mouse-adapted SARS-CoV-2
Source: Nat Commun. 2021 Sep 27;12:5654. doi: 10.1038/s41467-021-25903-x (PMC8476561; doi:10.1038/s41467-021-25903-x)
Supplement: Supplementary file 1 — Supplementary Information [file 41467_2021_25903_MOESM1_ESM.pdf]

Supplementary Materials for

Characterization and structural basis of a lethal mouse-adapted SARS-CoV-2

Shihui Sun[1†], Hongjing Gu[1†], Lei Cao[2†], Qi Chen[1†], Qing Ye[1†], Guan Yang[3†], Rui-Ting Li[1†], Hang Fan[1†],Yong-Qiang Deng[1], Xiaopeng Song[3], Yini Qi[3], Min Li[1], Jun Lan[2], Rui Feng[2], Yan Guo[1], Na Zhu[4], Si Qin[1], Lei Wang[2], Yi-Fei Zhang[1], Chao Zhou[1], Lingna Zhao[1], Yuehong Chen[1], Meng Shen[1], Yujun Cui[1], Xiao Yang[3], Xinquan Wang[5], Wenjie Tan[4], Hui Wang[1*], Xiangxi Wang[2*], Cheng-Feng Qin[1,6*]

Correspondence to: Cheng-Feng Qin (qincf@bmi.ac.cn), Xiangxi Wang (xiangxi@ibp.ac.cn), or Hui Wang (geno0109@vip.sina.com)

This PDF file includes:

Supplementary Figure 1 to 11

Supplementary Table 1 to 3

Supplementary References

Supplementary Figure 1

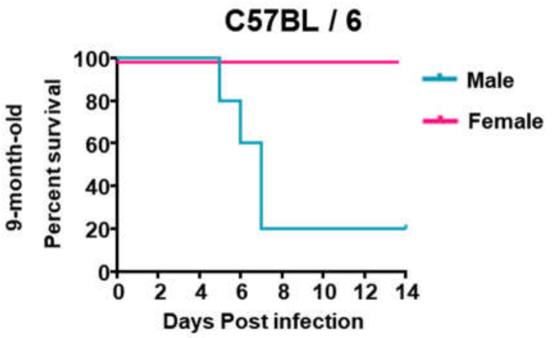

Supplementary Figure 1. Gender dependent mortality in MASCp36-infected C57BL/6 mice at age of 9 months. Survival curves of male and female C57BL/6 mice infected with 12,000 PFU of MASCp36 (n=5 per group).

.

Supplementary Figure 2

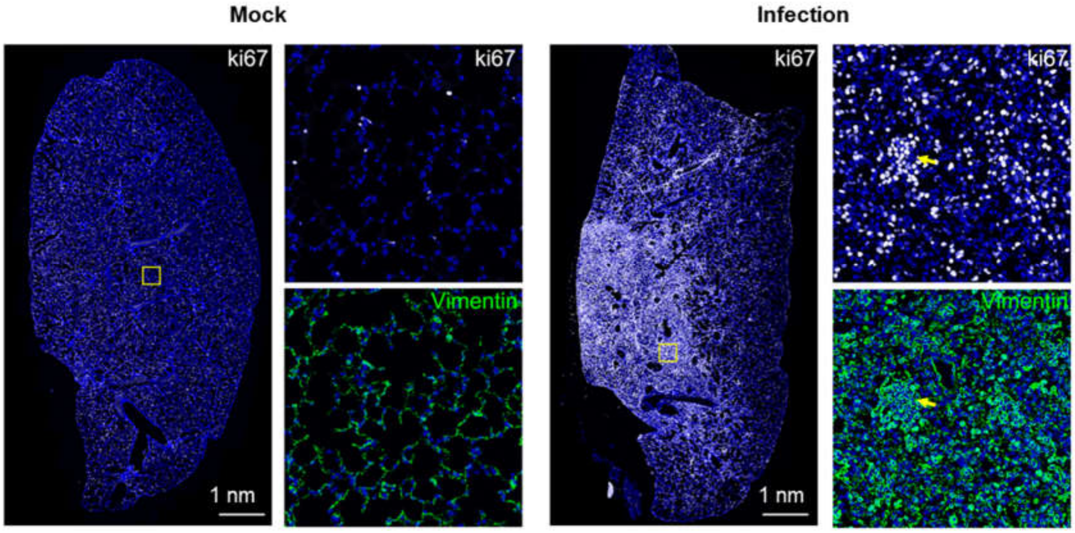

Supplementary Figure 2. MASCp36 infection causes proliferation of alveolar mesenchymal cells in lung of BALB/c mice. Multiplex immunofluorescence staining of lung sections from 9-month-old BALB/c mice infected with 120 PFU of MASCp36. The proliferation marker ki67 and mesenchymal marker Vimentin were shown in white and green, respectively. Framed areas are shown adjacently at higher magnifications. Arrows indicate proliferating alveolar mesenchymal cells (n=5 per group).

Supplementary Figure 3

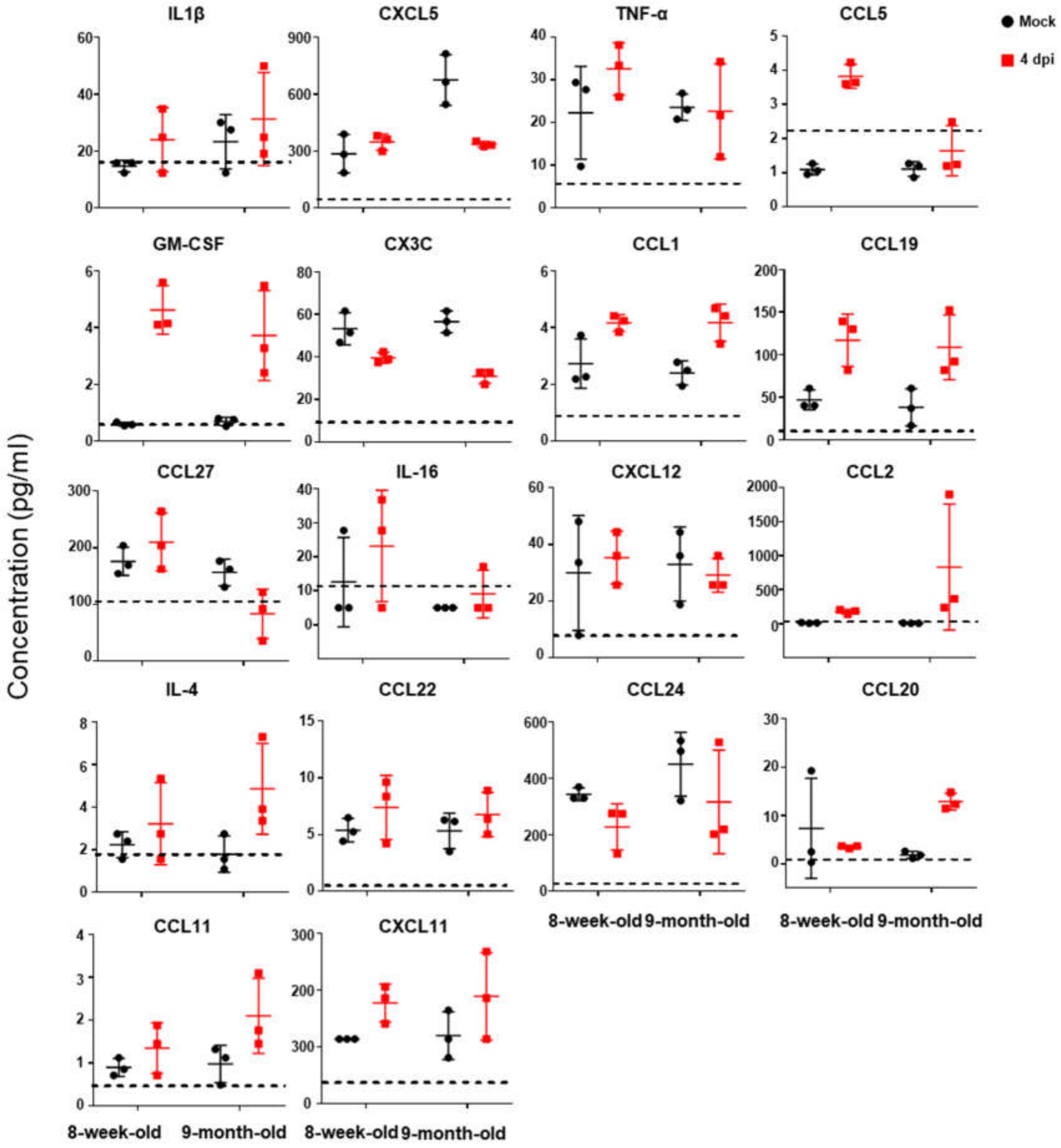

Supplementary Figure 3. Pulmonary cytokine and chemokine in mice infected with MASCp36. Male BALB/c mice (8-week-old and 9-month-old) were i.n. inoculated with 12,000 PFU of MASCp36, and lung homogenates were prepared at 4 dpi (n=3 per group). Cytokine and chemokine analysis was determined by Luminex and presented as mean±SEM. Dash lines denote the detection limit.

Supplementary Figure 4

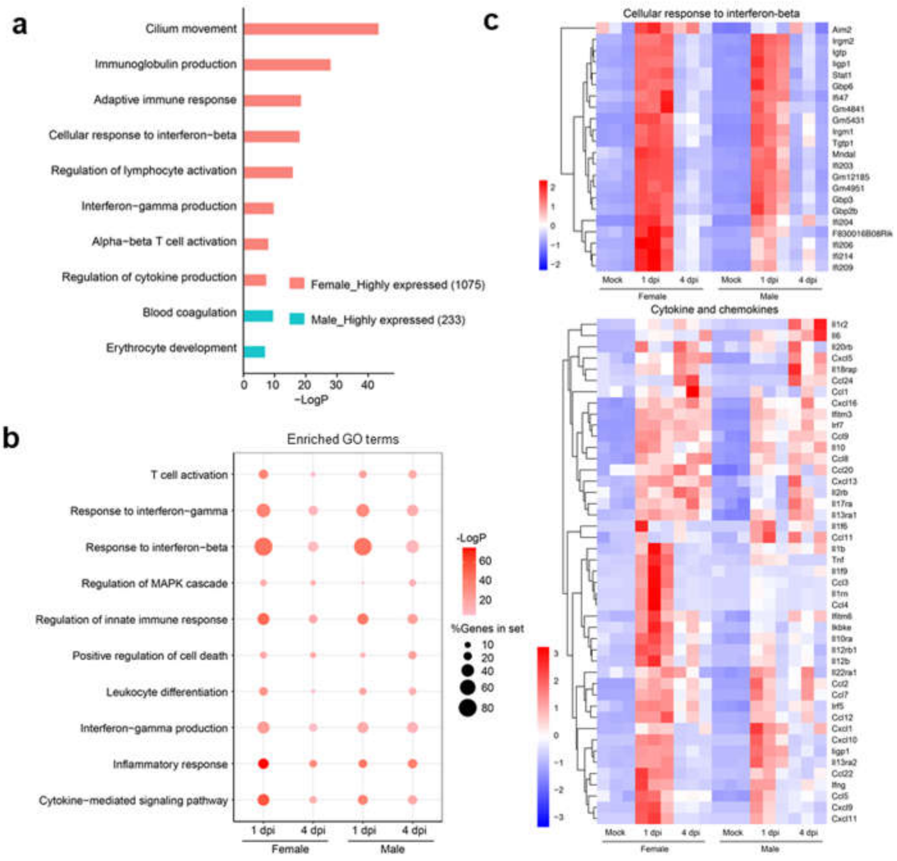

Supplementary Figure 4. Comparison of host transcriptional response to MASCp36 in lungs of female and male 9-month-old BALB/c mice. a Enriched GO terms of DEGs when comparing non-infected male and female transcriptome. **b** Dot plot visualization of enriched GO terms of up-regulated genes at 1 and 4 dpi. Gene enrichment analyses were performed using Metascape against the GO dataset for biological processes. The color of the dots represents the -LogP value for each enriched GO term, and size represents the percentage of genes enriched in each GO term. P-values in a and b were calculated based on accumulative hypergeometric distribution. **c** Heatmap indicating the expression patterns of genes belonging to GO annotation for "cellular response to interferon-beta" and 44 cytokine/chemokine genes. Colored bar represents Z-score of TPM.

Supplementary Figure 5

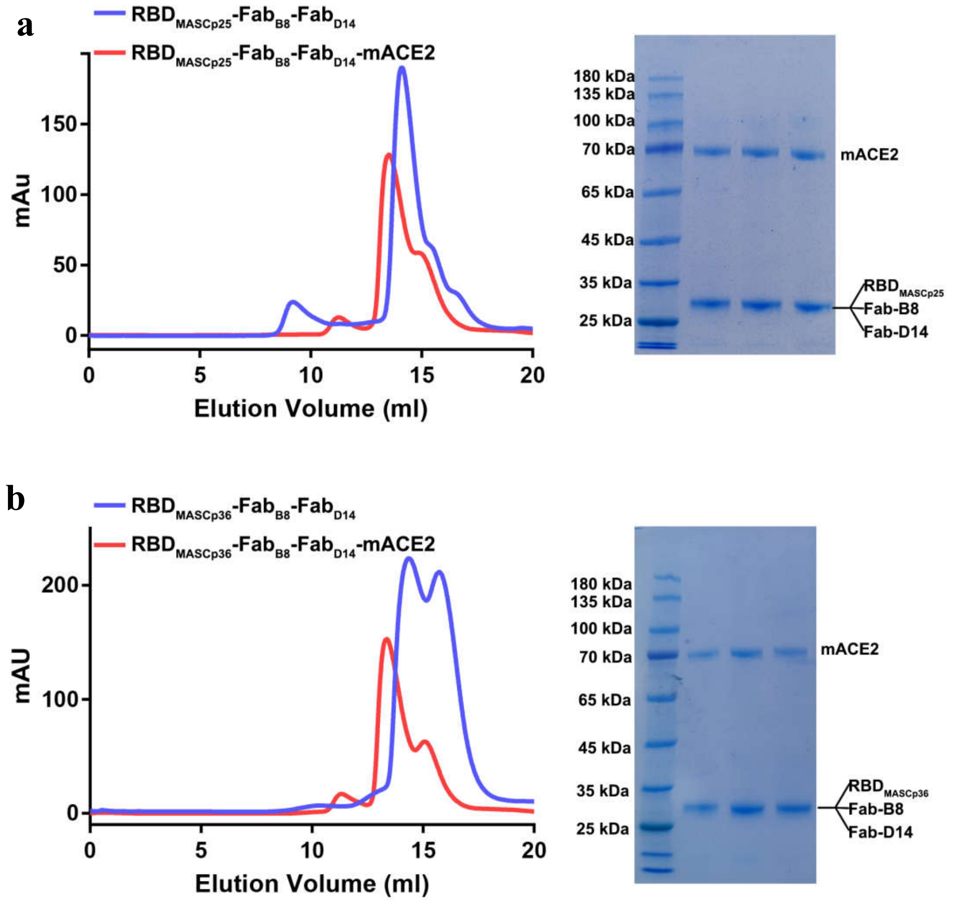

Supplementary Figure 5. Purification and characterization of RBD$_{MASCp25}$-Fab$_{B8}$-Fab$_{D14}$-mACE2 complex and RBD$_{MASCp36}$-Fab$_{B8}$-Fab$_{D14}$-mACE2 complex. a Gel filtration profiles of the ternary complex (RBD$_{MASCp25}$-Fab$_{B8}$-Fab$_{D14}$, colored by blue line) and quaternary complex (RBD$_{MASCp25}$-Fab$_{B8}$-Fab$_{D14}$-mACE2, colored by red line). SDS-PAGE analysis of the RBD$_{MASCp25}$-Fab$_{B8}$-Fab$_{D14}$-mACE2 complex. Three times repetitions in the experiments.**b** Gel filtration profiles of the ternary complex (RBD$_{MASCp36}$-Fab$_{B8}$-Fab$_{D14}$, colored by bule line) and quaternary complex (RBD$_{MASCp36}$-Fab$_{B8}$-Fab$_{D14}$-mACE2, colored by red line). SDS-PAGE analysis of the RBD$_{MASCp36}$-Fab$_{B8}$-Fab$_{D14}$-mACE2 complex. Three times repetitions in the experiments.

Supplementary Figure 6

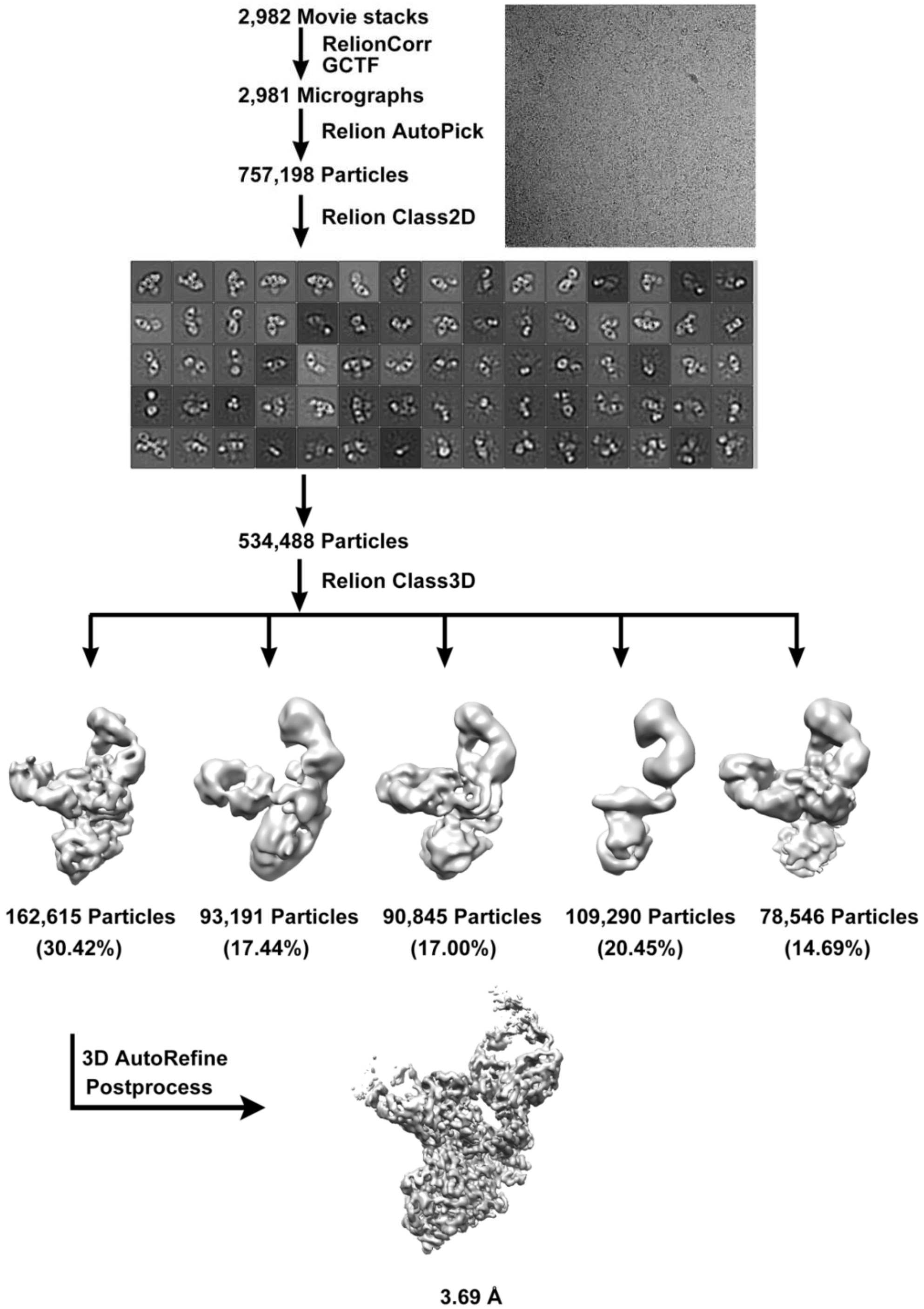

Supplementary Figure 6. Flow chart for the Cryo-EM data processing of $RBD_{MASCp36}$-Fab_{B8}-Fab_{D14}-mACE2 complex.

Supplementary Figure 7

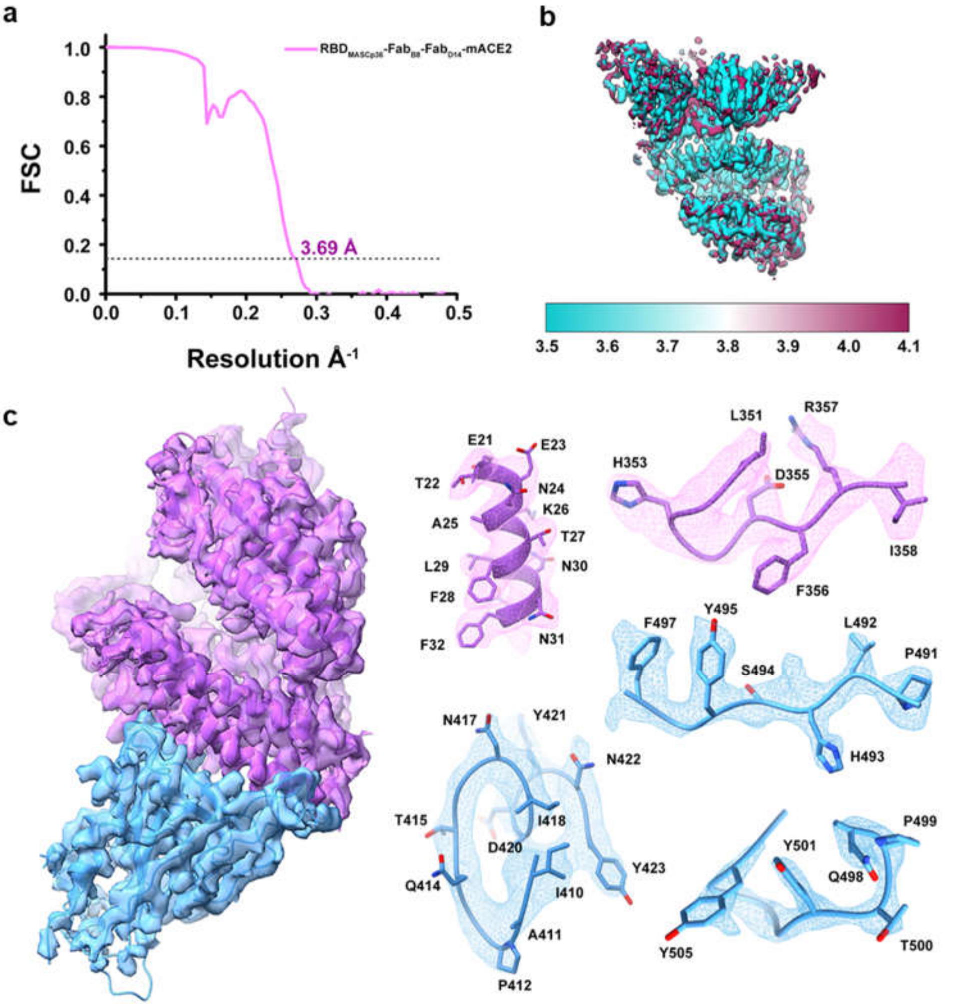

Supplementary Figure 7. Cryo-EM images and resolution evaluation of the EM map of RBD$_{MASCp36}$-Fab$_{B8}$-Fab$_{D14}$-mACE2 complex. a The gold-standard FSC curve of the final map. **b** Local resolution assessments of cryo-EM maps. Local-resolution evaluation of the map of the RBD$_{MASCp36}$-Fab$_{B8}$-Fab$_{D14}$-mACE2 complex using ResMap[1] is shown. **c** Electron density maps at the interface between the RBD$_{MASCp36}$ (colored in cyan) and mACE2 (colored in purple).

Supplementary Figure 8

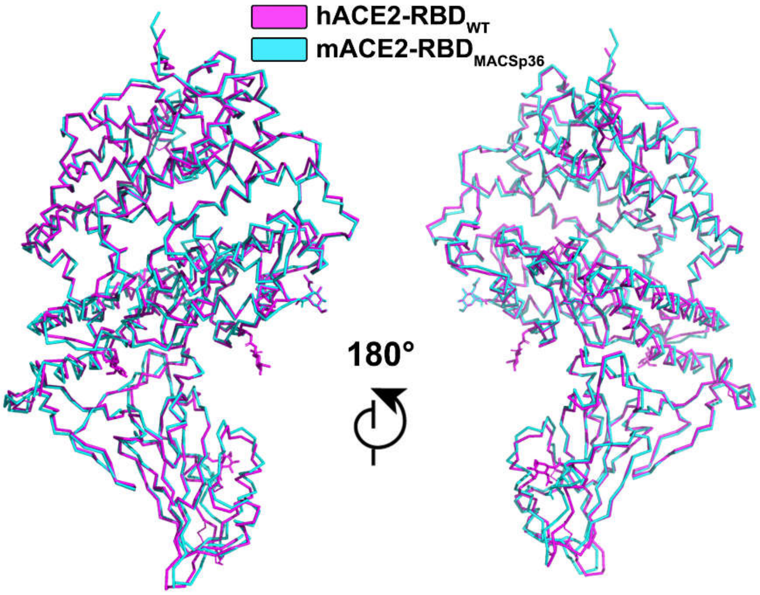

Supplementary Figure 8. Structural comparison of the mACE2-RBD$_{MACSp36}$ and hACE2-RBD$_{WT}$ complex. The mACE2-RBD$_{MACSp36}$ is shown in cyan, and the hACE2-RBD$_{WT}$ is shown in magenta.

Supplementary Figure 9

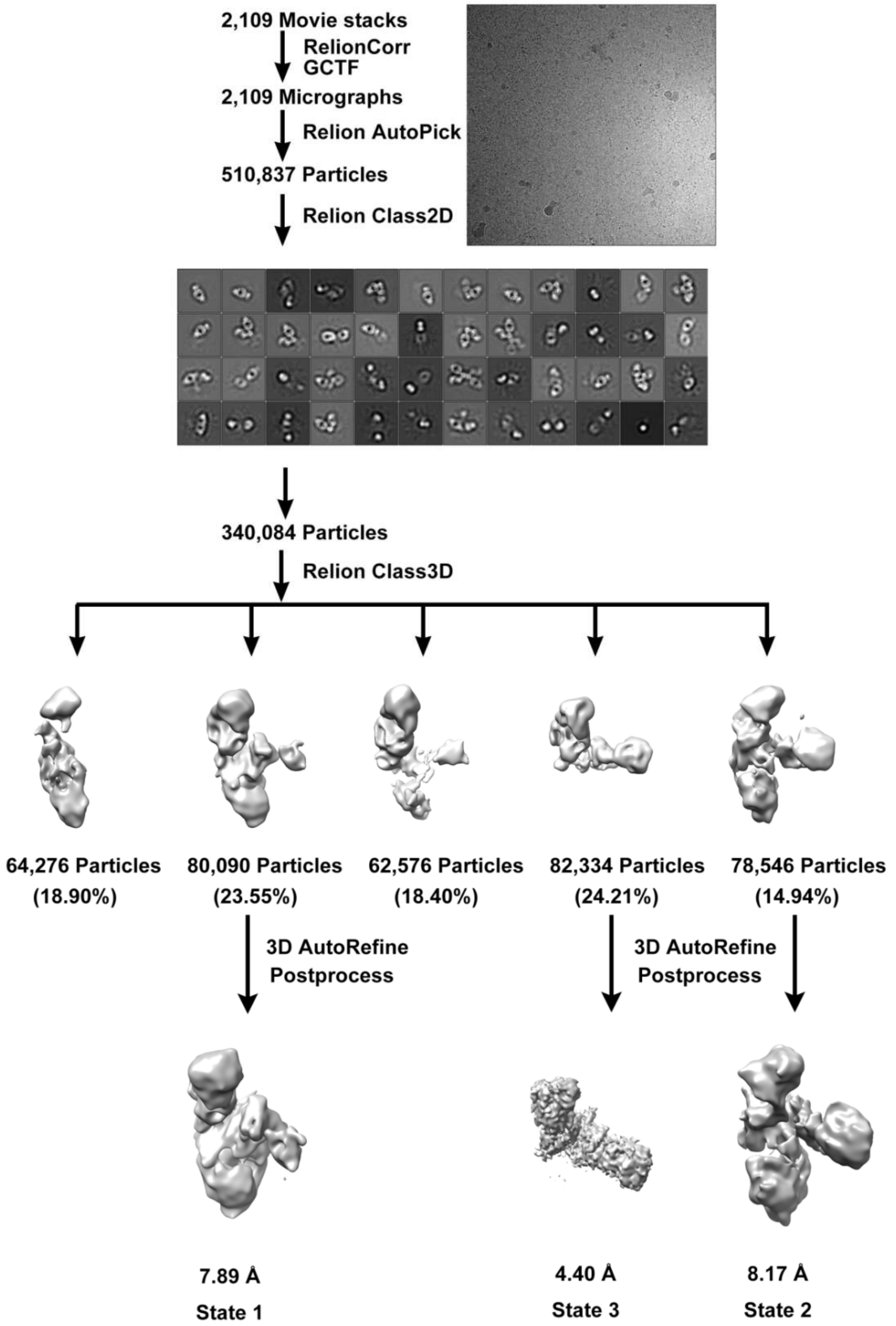

Supplementary Figure 9. Flow chart for Cryo-EM data processing of RBD$_{MASCp25}$-Fab$_{B8}$-Fab$_{D14}$-mACE2 complex.

Supplementary Figure 10

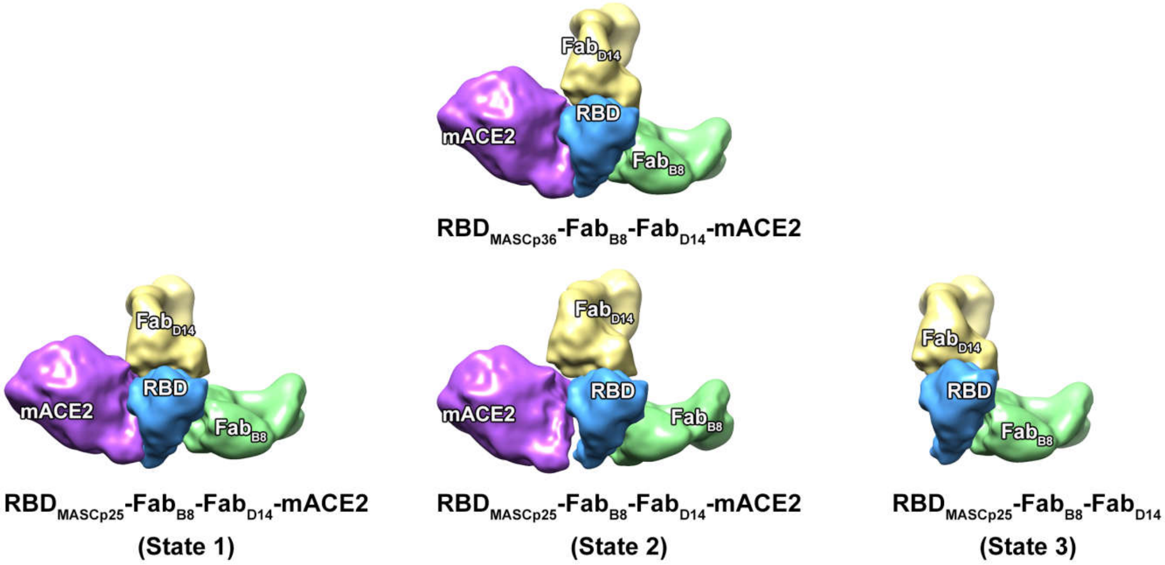

Supplementary Figure 10. Comparison of $RBD_{MASCp36}$-Fab_{B8}-Fab_{D14}-mACE2 complex with different conformations of $RBD_{MASCp25}$-Fab_{B8}-Fab_{D14}-mACE2 complex. All the subunits were filtered to a lower resolution at 15 Å. RBD mutant, mACE2, Fab_{B8} and Fab_{D14} are colored in blue, purple, yellow and green, respectively.

Supplementary Figure 11

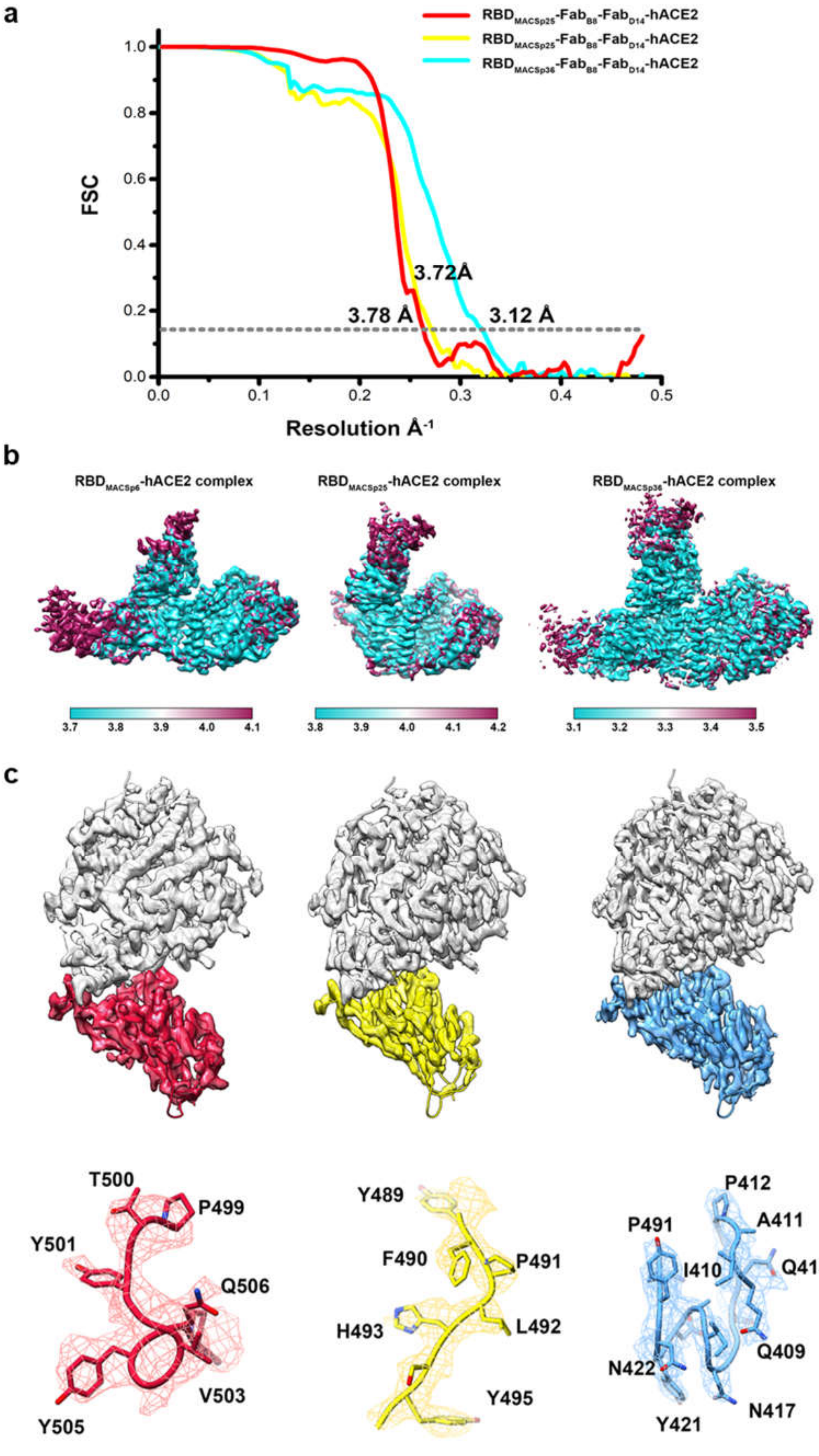

Supplementary Figure 11. Cryo-EM images and resolution evaluation of the EM

maps of RBD_{MACSp6}/$RBD_{MACSp25}$/$RBD_{MACSp36}$-Fab_{B8}-Fab_{D14}-hACE2 complex. a. The gold-standard FSC curves of the final maps. **b.** Local resolution assessments of cryo-EM maps. Local-resolution evaluation of the maps using ResMap[1] are shown. **c.** Electron density maps at the interface between the RBD and hACE2.

Supplementary table1. Cryo-EM data collection and atomic model refinement statistics of RBD mutant-mACE2 complex

Data collection and reconstruction statistics

Protein	$RBD_{MACSp36}$-Fab_{B8}-Fab_{D14}-mACE2	$RBD_{MACSp25}$-Fab_{B8}-Fab_{D14}-mACE2 (state1)	$RBD_{MACSp25}$-Fab_{B8}-Fab_{D14}-mACE2 (state2)	$RBD_{MACSp25}$-Fab_{B8}-Fab_{D14}-mACE2 (state3)
Voltage (kV)	300	300	300	300
Detector	K2	K2	K2	K2
Pixel size (Å)	1.04	1.04	1.04	1.04
Electron dose ($e^-/Å^2$)	60	60	60	60
Defocus range (μm)	1.25-2.7	1.25-2.7	1.25-2.7	1.25-2.7
Final particles	162,615	80,090	78,546	82,334
Resolution (Å)	3.69	7.89	8.17	4.40

Models refinement and validation statistics

Ramachandran statistics	
Favored (%)	92.38
Allowed (%)	7.43
Outliers (%)	0.11
Rotamer outliers (%)	0.09
R.m.s.d	
Bond lengths (Å)	0.02
Bond angles (°)	1.26

Supplementary table2. Residues of $RBD_{MACSp36}$ interacting with mACE2 at the binding interface (d < 4 Å)

$RBD_{MACSp36}$	mACE2
**Residues**	
N417	N30
	Q34
L455	Q34
F456	N31
A475	T27
F486	S82
N487	N24
Y489	T27
	F28
	N31
H493	N31
	E35
G496	D38
Q498	Q42
	L45
T500	Y41
	L45
	D355
	K357
Y501	Y41
	H353
	G354
G502	H353
	G354
Y505	H353

Supplementary table 3 Quantitative PCR primers which target the E subgenomic mRNA of SARS-CoV-2.

Target, Notation	Length	Primer Sequence (5' to 3')
E sgRNA-Leader	23	CGATCTCTTGTAGATCTGTTCTC
E sgRNA-Reverse	22	ATATTGCAGCAGTACGCACACA
E sgRNA-Probe	22	ACACTAGCCATCCTTACTGCGCTTCG

E subgenomic mRNA of SARS-CoV-2 designed by Wolfel R, *et al*[2]

Supplementary References:

1. Kucukelbir A, Sigworth FJ, Tagare HD. Quantifying the local resolution of cryo-EM density maps. *Nature methods* **11**, 63-65 (2014).
2. Wolfel R*, et al.* Virological assessment of hospitalized patients with COVID-2019. *Nature* **581**, 465-469 (2020).
