# Supplementary material for: Characterization and structural basis of a lethal mouse-adapted SARS-CoV-2
Source: Nat Commun. 2021 Sep 27;12:5654. doi: 10.1038/s41467-021-25903-x (PMC8476561; doi:10.1038/s41467-021-25903-x)
Supplement: Supplementary file 3 — Reporting Summary [file 41467_2021_25903_MOESM3_ESM.pdf]

nature portfolio

Corresponding author(s): Cheng-Feng Qin, Xiangxi Wang&Hui Wang

Last updated by author(s): Jul 18, 2021

Reporting Summary

Nature Portfolio wishes to improve the reproducibility of the work that we publish. This form provides structure for consistency and transparency in reporting. For further information on Nature Portfolio policies, see our Editorial Policies and the Editorial Policy Checklist.

Statistics

For all statistical analyses, confirm that the following items are present in the figure legend, table legend, main text, or Methods section.

n/a	Confirmed	
☐	☒	The exact sample size (n) for each experimental group/condition, given as a discrete number and unit of measurement
☐	☒	A statement on whether measurements were taken from distinct samples or whether the same sample was measured repeatedly
☐	☒	The statistical test(s) used AND whether they are one- or two-sided<br>*Only common tests should be described solely by name; describe more complex techniques in the Methods section.*
☒	☐	A description of all covariates tested
☐	☒	A description of any assumptions or corrections, such as tests of normality and adjustment for multiple comparisons
☒	☐	A full description of the statistical parameters including central tendency (e.g. means) or other basic estimates (e.g. regression coefficient) AND variation (e.g. standard deviation) or associated estimates of uncertainty (e.g. confidence intervals)
☒	☐	For null hypothesis testing, the test statistic (e.g. F, t, r) with confidence intervals, effect sizes, degrees of freedom and P value noted<br>*Give P values as exact values whenever suitable.*
☒	☐	For Bayesian analysis, information on the choice of priors and Markov chain Monte Carlo settings
☒	☐	For hierarchical and complex designs, identification of the appropriate level for tests and full reporting of outcomes
☒	☐	Estimates of effect sizes (e.g. Cohen's d, Pearson's r), indicating how they were calculated

Our web collection on statistics for biologists contains articles on many of the points above.

Software and code

Policy information about availability of computer code

Data collection	The details of data collection were described in Methods.
Data analysis	The details of data collection were described in Methods.

For manuscripts utilizing custom algorithms or software that are central to the research but not yet described in published literature, software must be made available to editors and reviewers. We strongly encourage code deposition in a community repository (e.g. GitHub). See the Nature Portfolio guidelines for submitting code & software for further information.

Data

Policy information about availability of data

All manuscripts must include a data availability statement. This statement should provide the following information, where applicable:

- Accession codes, unique identifiers, or web links for publicly available datasets
- A description of any restrictions on data availability
- For clinical datasets or third party data, please ensure that the statement adheres to our policy

The RNA-Seq data generated in this study have been deposited in the NCBI Gene Expression Omnibus (GEO) database under accession code GSE166778 （https://www.ncbi.nlm.nih.gov/geo/query/acc.cgi?acc=GSE166778）. The global cryo-EM maps of RBDMACSp6-FabB8-FabD14-hACE2, RBDMACSp25-FabB8-FabD14-hACE2, RBDMACSp36-FabB8-FabD14-hACE2 and RBDMACSp36-FabB8-FabD14-mACE2 complexes are deposited in the Electron Microscopy Data Bank (https://www.ebi.ac.uk/pdbe/emdb/) under accession numbers EMD-31542, EMD-31543, EMD-31544 and EMD-31546, respectively. The determined atomic models of RBDMACSp6-FabB8-FabD14-hACE2, RBDMACSp25-FabB8-FabD14-hACE2, RBDMACSp36-FabB8-FabD14-hACE2 and RBDMACSp36-FabB8-FabD14-mACE2 complexes have been deposited to the Protein Data Bank (https://www.rcsb.org/) under accession codes 7FDG, 7FDH, 7FDI, 7FDK respectively. All the high throughput sequencing data related have been deposited in the Sequence Read Archive (SRA, https://www.ncbi.nlm.nih.gov/sra) of National Center of Biotechnology

Information (NCBI) , with the BioProject accession number of PRJNA746117.

Field-specific reporting

Please select the one below that is the best fit for your research. If you are not sure, read the appropriate sections before making your selection.

- [x] Life sciences
- [ ] Behavioural & social sciences
- [ ] Ecological, evolutionary & environmental sciences

For a reference copy of the document with all sections, see nature.com/documents/nr-reporting-summary-flat.pdf

Life sciences study design

All studies must disclose on these points even when the disclosure is negative.

Sample size	Sample size was estimated on the basis of similar research reported in the literature.
Data exclusions	No data were excluded.
Replication	Data were performed with technical replications.
Randomization	No randomization was used.
Blinding	No Blinding was used.

Reporting for specific materials, systems and methods

We require information from authors about some types of materials, experimental systems and methods used in many studies. Here, indicate whether each material, system or method listed is relevant to your study. If you are not sure if a list item applies to your research, read the appropriate section before selecting a response.

Materials & experimental systems

n/a	Involved in the study	
[ ]	[x]	Antibodies
[ ]	[x]	Eukaryotic cell lines
[x]	[ ]	Palaeontology and archaeology
[ ]	[x]	Animals and other organisms
[x]	[ ]	Human research participants
[x]	[ ]	Clinical data
[x]	[ ]	Dual use research of concern

Methods

n/a	Involved in the study	
[x]	[ ]	ChIP-seq
[x]	[ ]	Flow cytometry
[x]	[ ]	MRI-based neuroimaging

Antibodies

Antibodies used	The details of antibodies used were in Methods.
Validation	Validation data about the antibodies obtained from commercial sources are available on the manufacturer's website and/or data sheet.

Eukaryotic cell lines

Policy information about cell lines

Cell line source(s)	HEK Expi 293F cells were obtained commercially from the Gibco(ThermoFisher,A14527)
Authentication	HEK Expi 293F cells were authenticated by the Gibco .
Mycoplasma contamination	We already confirmed that the all cell lines tested negative for mycoplasma contamination.
Commonly misidentified lines (See ICLAC register)	None.

Animals and other organisms

Policy information about studies involving animals; ARRIVE guidelines recommended for reporting animal research

Laboratory animals	BALB/c and C57/BJ, Female and Male, 9months and 8weeks.
Wild animals	None
Field-collected samples	The detail were describe in Methods.
Ethics oversight	The Animal Experiment Committee of Laboratory Animal Center, Beijing Institute of Microbiology and Epidemiology approval number: IACUC-DWZX-2020-002

Note that full information on the approval of the study protocol must also be provided in the manuscript.
